# Analysis of the different growth years accumulation of flavonoids in *Dendrobium moniliforme* (L.) Sw. by the integration of metabolomic and transcriptomic approaches

**DOI:** 10.3389/fnut.2022.928074

**Published:** 2022-09-26

**Authors:** Yingdan Yuan, Jiajia Zuo, Hanyue Zhang, Mengting Zu, Sian Liu

**Affiliations:** College of Horticulture and Plant Protection, Yangzhou University, Yangzhou, China

**Keywords:** *Dendrobium moniliforme*, transcriptome, metabolome, flavonoids, different growth years

## Abstract

*Dendrobium moniliforme* (L.) Sw. is a valuable herbal crop, and flavonoids are primarily distributed as active ingredients in the stem, but the composition and synthesis mechanisms of flavonoids in different growth years are not clear. The accumulation of flavonoids in *D. moniliforme* from four different years was investigated, using a combined metabolomics and transcriptomics approach in this study. The phenylpropanoid and flavonoid biosynthetic pathways were significantly enriched in the Kyoto Encyclopedia of Genes and Genomes (KEGG) enrichment analysis of differentially expressed genes (DEGs) and differentially accumulated metabolites (DAMs). The widely targeted metabolomics technique revealed a total of 173 kinds of flavonoid metabolites. The metabolomics data confirmed the trend of total flavonoids (TF) content in stems of *D. moniliforme*, with chalcone, naringenin, eriodictyol, dihydroquercetin, and other flavonoids considerably up-accumulating in the third year. Twenty DEGs were detected that regulate flavonoid synthesis and the expression of these genes in different growth years was verified using real-time quantitative PCR (qRT-PCR). Furthermore, a comprehensive regulatory network was built for flavonoid biosynthesis and it was discovered that there is one *FLS* gene, one *CCR* gene and two MYB transcription factors (TFs) with a high connection with flavonoid biosynthesis by weighted gene co-expression network analysis (WGCNA). In this study, the correlation between genes involved in flavonoid biosynthesis and metabolites was revealed, and a new regulatory mechanism related to flavonoid biosynthesis in *D. moniliforme* was proposed. These results provide an important reference for the farmers involved in the cultivation of *D. moniliforme*.

## Introduction

*Dendrobium* is an epiphytic orchid whose stems are commonly used for the generation of nutritional beverages and food raw material (1). It has the effects of nourishing the kidney, hydrating the lung, benefitting the stomach; therefore, it is a type of plant with high development and utilization value (2). In the past, *Dendrobium moniliforme* (L.) Sw. was commonly employed as an attractive plant (3). The distribution of wild *D. moniliforme* is mainly in tropical and subtropical regions of Asia, such as China, Japan, South Korea, and Myanmar (4). In recent years, many studies have revealed that *D. moniliforme* also has medicinal value, including anti-inflammatory and antioxidant properties (5). Many studies have demonstrated that *D. moniliforme* includes a wide range of beneficial secondary metabolites, such as alkaloids (6) and flavonoids (7). The flavonoids in *D. moniliforme* are an important part of its pharmacological activity and an important index for evaluating *D. moniliforme* quality. Flavonoids are important secondary metabolites in plants, which have been proven to exert healthy functions in the human body and to have significant pharmacological effects such as anti-inflammatory, immunosuppressive, and antioxidant (8). Researchers discovered that the amount and composition of flavonoids change between species according to factors such as habitat, growth stage, and tissues (9). Therefore, due to the diversity of flavonoids and the complex laws of their distribution in different plants, it is significant to explore the flavonoid composition and biosynthetic pathways of *D. moniliforme* for different growth years.

As perennial plants, the content of medicinal components in *Dendrobium* spp. varies with the year of harvesting. Therefore, the active components in medicinal plants of different growth years should be evaluated in order to obtain high quality and optimal benefits of the herbs. At present, the most studies on the harvesting period of *Dendrobium* are reported on *D. officinale*, *D. huoshanense* and *D. nobile*; however there are few reports on *D. moniliforme* (10). People have greatly overlooked the potential of *D. moniliforme* as a medicinal plant. In summary, in this study, the *D. moniliforme* stems were used as the research object, and the transcriptome combined with the metabolome was analyzed so as to select the key genes that might affect the flavonoid synthesis in *D. moniliforme*. Some DEGs involved in flavonoid synthesis were also selected for real-time quantitative PCR (qRT-PCR) to prove the reliability of transcriptome data and further analysis. In addition, weighted gene co-expression network analysis (WGCNA) was used to obtain modules with high correlation of flavonoids, and screened the key genes involved in flavonoid biosynthesis. The data obtained in this study will provide important information for future research on the accumulation of flavonoids in *D. moniliforme* and provide a theoretical basis for determining the ideal harvesting year of *D. moniliforme*.

## Materials and methods

### Plant materials and determination of total flavonoids and alkaloids

*Dendrobium moniliforme* (L.) Sw. were cultivated artificially and gathered in the greenhouse of Anhui Tongjisheng Biotechnology Co., Ltd. In terms of culture conditions, the samples were consistent with previous research (11). The stems of *D. moniliforme* were collected from 1-year-old (Stem length: 17.35 ± 3.87 cm; stem diameter: 1.98 ± 0.54 cm), 2-year-old (Stem length: 22.47 ± 2.59 cm; stem diameter: 2.33 ± 0.48 cm), 3-year-old (Stem length: 27.7 ± 6.92 cm; stem diameter: 2.67 ± 0.52 cm), and 4-year-old (Stem length: 30.45 ± 3.58 cm; stem diameter: 3.64 ± 1.32 cm) plants, respectively, as the research object (Supplementary Figure 1). We removed the leaves and partial roots from the stems to get the clean stems. To extract ribonucleic acid (RNA) and metabolites, all materials were frozen in liquid nitrogen at -80^°^C. To determine the total alkaloids (TA) and total flavonoids (TF), all materials were washed and dried. Furthermore, in this study, all experiments were carried out in three biological replicates.

TF and TA were extracted and measured using a plant flavonoid kit and a plant alkaloid kit (Suzhou Comin Biotechnology Co., Ltd., Suzhou, China), respectively.

### Widely targeted metabolomics profiling

The frozen samples (systematic samples maintained in a temperature of -80^°^C) were crushed with a zirconia head at 30 Hz for 15 min using a blender mill (MM 400, Retsch). The powder was then weighed and extracted overnight at 4^°^C with 1.0 mL of 70% aqueous methanol. The supernatant was collected and filtered (microporous membrane filters with pore sizes of 0.22 μm) before LC-ESI-MS/MS analysis after centrifugation at 10,000 g for 10 min. An LC-ESI-MS/MS system was used to examine the sample extracts (HPLC: Shim-pack UFLC Shimadzu CBM30A system^1^; MS: Applied Biosystems 6500 Q TRAP).^2^ Qualitative and quantitative mass spectrometry analysis of metabolites in samples was based on the KEGG compound database, the MetWare database (MWDB), and multiple reaction monitoring (MRM). Metabolite identification is based on the accurate mass of metabolites, MS2 fragments, MS2 fragment isotope distribution and retention time (RT). The secondary spectrum and RT of the Metware company’s database are intelligently matched one by one, and the MS tolerance and MS2 tolerance are set to 20 and 20 ppm, respectively. For each treatment group, three biological replicates were examined individually. The samples were evaluated using Yang’s methods under the following HPLC conditions (12).

Principal component analysis (PCA) was used to investigate the specific accumulation of *D. moniliforme* metabolites in different growth years using R.^3^ The data were normalized, and all samples were examined using a cluster heatmap, which was then generated. The following conditions were used to screen differentially accumulated metabolites (DAMs): foldchange ≥ 2 and foldchange ≤ 0.5, VIP ≥ 1, and the up and down regulation of differential metabolites was compared between different comparison groups. The mean values of the relative content of the differential metabolites in each group were standardized by z-score and then subjected to K-means clustering analysis to analyze trends in the relative content of metabolites in distinct subgroups.

### Ribonucleic acid extraction, Illumina sequencing, and differentially expressed genes analysis

According to the manufacturer’s instructions, total RNA of *D. moniliforme* was extracted using the OmniPlantRNA kit (CWBIO, China), mRNA libraries of each sample were constructed, and the libraries were sequenced using Illumina platform. RNA extraction, library construction and sequencing were carried out as described by Yang’s method (12). Using the HISAT2 software, the filtered reads were mapped to the reference genome (13).^4^ As a measure of transcription or gene expression, fragments per kilobase of transcript per million mapped reads (FPKM) were utilized. The DESeq2 was used to find differentially expressed genes (DEGs) (14), and the filter condition was | log2(fold change)| > 1, with *p*-value < 0.05. TopGO and clusterprofiler were used to enrich all DEGs in Gene Ontology (GO) and the Kyoto Encyclopedia of Genes and Genomes (KEGG) to better understand the function and critical pathway of DEGs.

### Gene co-expression network construction

WGCNA was conducted by using R software package. Before constructing the network, the RNA sequencing data was examined to eliminate low-quality genes and low-quality samples. Then, the Pearson correlation coefficients of all DEGs were calculated and the appropriate soft threshold ß was automatically selected (15). The Pearson result weighted by ß exponent was transformed into adjacency matrix (16). Then, the adjacency matrix was transformed into topological overlap (TOM) matrix and TOM was used to demonstrate the similarity expression of genes. Finally, hierarchical clustering method is used to generate a hierarchical clustering tree of DEGs, and similar modules are combined (17). Hub genes are commonly used for highly connected genes, which have a high level of connectivity in the co-expression module. Depending on the size of the module, the top 20 genes with the strongest connection have been designated as hub genes, and genes were further examined in these modules. Cytoscape (v.3.6.1) was used to build and visualize a network of gene-gene interactions.

### Integration analysis of transcriptome and metabolome

According to the metabolite content and gene expression value in the stem of *D. moniliforme* at different growth years, the DEGs and the DAMs of flavonoid biosynthesis pathway in each comparison group were analyzed. First, pathway analysis was used to analyze the DEGs and DAMs related to flavonoid biosynthesis. Moreover, in order to better understand the relationship between transcriptome and metabolome, DEGs and DAMs were mapped to the KEGG pathway database to obtain their common pathway information.

### Real-time quantitative PCR validation

Eleven DEGs were screened for qRT-PCR using specific primers designed by Oligo7 software. Supplementary Table 1 lists the primers that were used in this study. qRT-PCR was performed on the ABI 7500 Real-time PCR system (Applied Biosystems) according to the manufacturer’s instructions. 2^–ΔΔCT^ method was used for relative quantitative analysis of the data, and the internal reference gene was *Actin* (18). Three replicates were analyzed for each sample to ensure reproducibility and reliability (Supplementary Figure 2).

### Statistical analysis

The differences between multiple groups were analyzed using a one-way ANOVA followed by Duncan’s multiple comparisons test. The experimental data were expressed as the mean ± standard deviation, *p*-values less than 0.05 were regarded as statistically significant. SPSS 22.0 software was used for statistical analysis and GraphPad Prism 8.0 software was used for drawing. In this study, all experiments were conducted in three biological replicates.

## Results

### Measurement of total flavonoid and alkaloid contents in *Dendrobium moniliforme*

**FIGURE 1:**
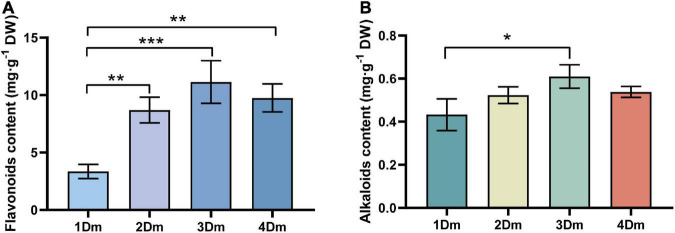
Determination of total flavonoids and alkaloids in the stem of *D. moniliforme*. **(A)** Content of total flavonoids in stems of *D. moniliforme* with different growth years; **(B)** contents of total alkaloids in stems of *D. moniliforme* with different growth years. The asterisk “*” indicates statistical differences in the same indicator between different growth years by a *t*-test, with a significant difference of *p* < 0.05 (*p < 0.05, **p < 0.01, ***p < 0.001). Error bars represent standard deviations (*n* = 3).

In order to determine the accumulation of flavonoids and alkaloids in the stems of *D. moniliforme* in different years, their contents in different growth years were measured. In general, the contents of flavonoids were very variable at different growth stages. As seen in the Figure 1, the flavonoid content increased from 1Dm to 3Dm, peaked at 3Dm then dropped in the fourth year. The highest flavonoid in the stem of *D. moniliforme* was 11.13 mg^.^g^–1^ DW and the lowest 3.35 mg^.^g^–1^ DW (Figure 1). The flavonoid content of 2-year-old, 3-year-old, and 4-year-old *D. moniliforme* was significantly different from that of 1-year-old *D. moniliforme*. The trend of the content of alkaloids in stems of *D. moniliforme* in four different growth years was similar to that of the contents of flavonoids. The alkaloid content increased in the first 3 years, reached the maximum value of 0.61 mg^.^g^–1^ DW in the third year, and decreased in the fourth year (Figure 1). They are all significantly higher than that in the first year. The results suggested that the third year of *D. moniliforme* growth could be an essential stage of accumulation of flavonoids and alkaloids.

### Metabolite profiling of different growth years of *Dendrobium moniliforme*

In order to explore the metabolic changes during the different growth years of *D. moniliforme*, metabolic analysis on the stems of *D. moniliforme* with four growth years was carried out by using widely targeted metabolomics. 767 metabolites were identified from 1Dm, 2Dm, 3Dm and 4Dm, indicating that the spectrum of *D. moniliforme* metabolites was diverse in different growth years. Supplementary Tables 2–7 listed all the metabolites identified in all samples. PCA was used to evaluate 12 samples in order to gain a preliminary understanding of the overall metabolic difference. The analytical results show that there are significant differences between each group, but no significant differences within the group (Supplementary Figure 3A). The biological repeats were all gathered together, indicating that the metabolomic data is highly reliable. The heatmap results revealed that flavonoids accumulated at a high level in 3 and 4Dm (Supplementary Figure 3B). The maximum expression of organic acid was found in 2Dm, compared with the samples of other years. Furthermore, lipids, amino acids and derivatives, alkaloids, nucleotides and derivatives, quinones and terpenoids were more abundant in 2Dm and 4Dm than in 1Dm and 3Dm.

A K-means cluster analysis was performed on 366 metabolites in four developmental stages. The nine figures showed obvious cluster changes of metabolites, and the change trend is shown in Supplementary Figure 3C. Furthermore, these 366 metabolites contained 63 flavonoids. Notably, 61.9% of TF were found in Clusters 4, Cluster 7 and Cluster 8. Interestingly, in the metabolite expression trends of these three clusters, the levels of expression of metabolites in 3 and 4Dm were higher than in 1 and 2Dm. A total of 480 DAMs were identified (Supplementary Figure 3D). The most up-accumulated DAMs were found in 1Dm vs. 4Dm, the least up-accumulated DAMs were found in 3Dm vs. 4Dm. While 2Dm vs. 3Dm had the most down-regulated DAMs, 3Dm vs. 4Dm had the least down-regulated DAMs. According to Supplementary Figure 3D, the highest number of DAMs was found in 1 and 2Dm, while the lowest number of DAMs was found in 3Dm vs. 4Dm. In Supplementary Figure 4, the top 10 up-accumulated and down-accumulated metabolites with significant differences were listed.

### Ribonucleic acid sequencing and identification of differentially expressed genes

The RNA-Seq data were submitted to NCBI with accession number PRJNA776418. Transcriptome analysis was used to identify DEGs in stems to better understand the molecular basis of *D. moniliforme*. The DEGs among different comparison groups of *D. moniliforme* can be seen using the volcano figure (Supplementary Figure 5). There were 3,734 DEGs in six comparison groups (1Dm vs. 2Dm, 1Dm vs. 3Dm, 1Dm vs. 4Dm, 2Dm vs. 3Dm, 2Dm vs. 4Dm, 3Dm vs. 4Dm) using | Log_2_FC| > 1 and *p*-value < 0.05 as screening criterion. The largest number of DEGs were discovered in 2Dm vs. 3Dm (2,183). The lowest amount of DEGs were found in 3Dm vs. 4Dm (228).

### Gene ontology and Kyoto encyclopedia of genes and genomes enrichment of differentially expressed genes

The functions of the DEGs were categorized according to the classification of the GO database. As shown in Supplementary Figure 6, “Biosynthetic processes” was the most enriched subcategory in the biological process (BP) category, followed by “Metabolic processes.” The most enriched subcategories in the molecular function (MF) category were “Transferase activity” and “Transporter activity.” DEGs are typically found in “Membranes” and “Cell walls” in the cell component (CC) category.

To understand the biological functions of DEGs, the transcriptome sequencing data were blasted to the KEGG database (Supplementary Figure 7). Among the top 20 enriched pathways, the largest proportion of DEGs was located in the “Metabolism pathway,” while “Environmental information processing,” and “Organismal systems” were in the second and third place. Among them, “Phenylpropanoid biosynthesis” and “Starch and sucrose metabolism” were significantly enriched in all comparison groups, except for 3Dm vs. 4Dm. “Fructose and mannose metabolism” was significantly enriched in both the 1Dm vs. 3Dm and 2Dm vs. 4Dm. In both 1Dm vs. 3Dm and 3Dm vs. 4Dm, “Tropane, piperidine and pyridine alkaloid biosynthesis” were remarkably enriched. In Supplementary Figure 8, the top 20 KEGG pathways are listed.

KEGG enrichment analysis showed that many DEGs are associated with metabolic pathways. Secondary metabolism-related pathways are important in medicinal plants. In this study, secondary metabolism-related DEGs in 1Dm vs. 2Dm, 1Dm vs. 3Dm, 2Dm vs. 4Dm, 3Dm vs. 4Dm, and 3Dm vs. 4Dm were classified as 34, 49, 47, 56, 57, and 11 secondary-metabolic KEGG pathways. Among them, 56, 57, and 11 ts. In this study, meivatives, quiee gene was e appropriate soft threshold ractfour growth years of *D. moniliforme*.

### Identified metabolites and genes involved in flavonoid biosynthesis pathway

**FIGURE 2:**
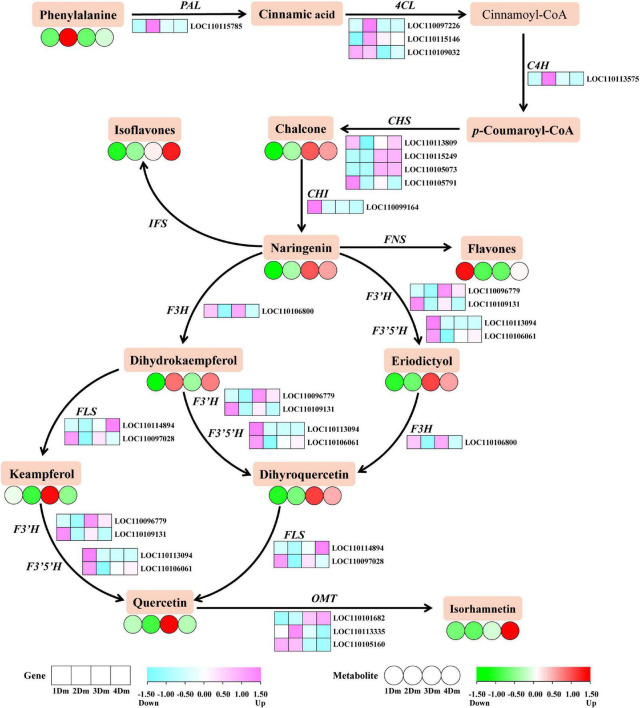
The flavonoids biosynthetic pathway of *D. moniliforme*. The heatmap shows the expression patterns of DEGs and DAMs during flavonoid biosynthesis. The rectangles represent the expression changes of DEGs and the circles represent the expression changes of DAMs. The color scale indicates the relative amounts of DEGs and DAMs. Darker colors indicate higher expression. Pink represents up-regulated DEGs, and blue represents down-regulated DEGs. Red represents up-regulated DAMs and green represents down-regulated DAMs. Key enzyme gene abbreviation: C4H, cinnamate 4-hydroxylase; PAL, phenylalanine ammonia lyase; 4CL, 4-coumarate: CoA ligase; CHS, chalcone synthase; CHI, chalcone isomerase; F3H, flavonoid 3-hydroxylase; F3’H, flavonoid 3’-hydroxylase; FLS, flavonol synthase; IFS: isoflavone synthase; FNS, flavone synthase; OMT, O-methyltransferase.

As shown in Figure 2, there have been identified a total of 20 DEGs involving the flavonoid biosynthetic pathway. Overall, the levels of expression of two *CHS* genes (*LOC110105249*, *LOC110105073*), one *F3’H* gene (*LOC110096779*), one *FLS* gene (*LOC1100114984*), and one OMT gene (*LOC110101682*) were higher in 3Dm or 4Dm than in 1 and 2Dm. However, the expression of some genes upstream of the flavonoid biosynthetic pathway was higher in 1 and 2Dm than in 3Dm or 4Dm, such as *PAL*, *4CL* and *C4H*. In order to verify the reliability of transcriptomic results, 17 DEGs involved in flavonoid biosynthesis were further selected for qRT-PCR analysis. qRT-PCR results were basically consistent with RNA-seq results, indicating the validity of RNA-seq results (Supplementary Figure 2G).

There have been identified a total of 34 DAMs related to the flavonoid biosynthetic pathway. The DAMs content revealed diverse trends of expression each year, but most metabolites were most expressive at 3 and 4Dm, except keampferol, dihydrokaempferol and flavones. In addition, keampferol was more expressed in 1 and 3Dm than 2 and 4Dm, while dihydrokaempferol was exactly the opposite of keampferol; that is, it was more expressed in 2 and 4Dm than 1 and 3Dm.

### Gene co-expression network analysis

**FIGURE 3:**
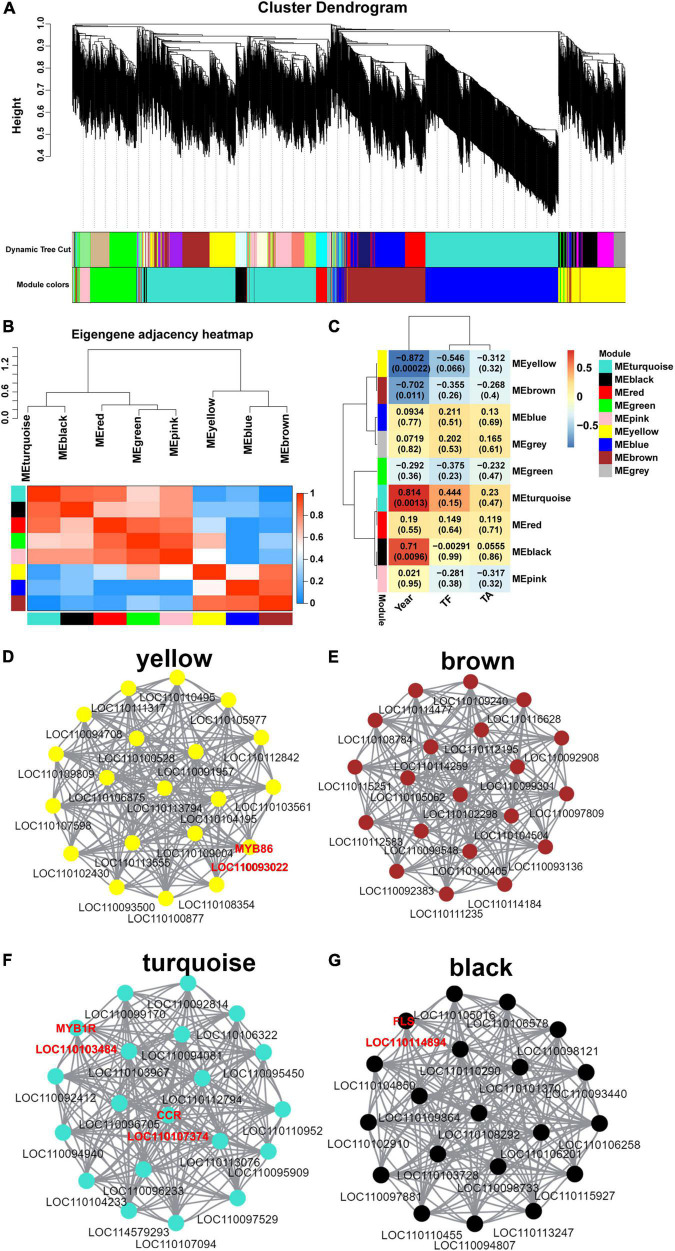
Weighted gene co-expression network of *D. moniliforme*. **(A)** Clustering dendrogram of DEGs, with dissimilarity based on the topological overlap, together with assigned module colors. The clustered branches represent different modules, and each line represents one DEG. **(B)** The heatmap of connectivity of eigengenes. **(C)** Module-trait associations. Each row corresponds to a module characteristic gene (eigengene), and each column corresponds to a trait. Each cell contains a corresponding correlation coefficient and *p*-value. **(D–G)** *D. moniliforme* transcriptome co-expression network diagram: **(D)** Yellow module; **(E)** brown module; **(F)** turquoise module; **(G)** black module.

To investigate the gene regulatory network of flavonoid synthesis in *D. moniliforme* stems, co-expression analysis and network construction for 3,734 DEGs were performed. The analysis yielded nine different modules (black, blue, brown, green, gray, pink, red, turquoise and yellow) in a dendrogram, where modules are clusters of highly correlated genes that are co-expressed within the same module (Figure 3A). To detect the interactions between gene models, network heatmap for co-expression modules was performed. The heatmap of the co-expression network is in red, indicating high DEG co-expression within the module and low co-expression outside the module (Figure 3B). The modules associated with TF, TA, and different growth years (Year) were identified from the above modules (Figure 3C). The results showed that four modules were highly correlated with TF, TA, and Year, including yellow, brown, turquoise and black gene modules, while the rest of the modules were less correlated with TF, TA, and Year. Among them, turquoise and black gene modules were significantly positively correlated with Year, and yellow and brown gene modules were significantly negatively correlated with Year.

In the co-expression networks, hub genes are highly co-expressed with other genes and play important roles in key pathways. 20 hub genes were identified in each of the yellow, brown, turquoise, and black modules (Figures 3D–G), and these hub genes were highly connected. In Supplementary Figure 9, a heatmap of the expression of hub genes in the four modules was performed. In both turquoise and black modules, hub genes had high expression in 3Dm and 4Dm. The expression of hub genes in the yellow and brown modules was higher in 1Dm and 2Dm than in 3Dm and 4Dm. Overall, this result was generally consistent with the variation of flavonoid and alkaloid contents in *D. moniliforme*. In-depth analysis revealed that the black module identified one *FLS* gene (*LOC110114894*) involved in flavonoid biosynthesis, which had strong connectivity with other hub genes, which further indicated that *FLS* genes are key genes in flavonoid biosynthesis. In yellow and turquoise modules, two MYB transcription factors (TFs) (*LOC110103484* and *LOC110093022*) regulating flavonoid synthesis were found. In addition, in the turquoise module, a key enzyme *cinnamoyl-CoA reductase* (*CCR*, *LOC110107374*) involved in lignin synthesis was identified.

### Integrated metabolomic and transcriptomic analysis

**FIGURE 4:**
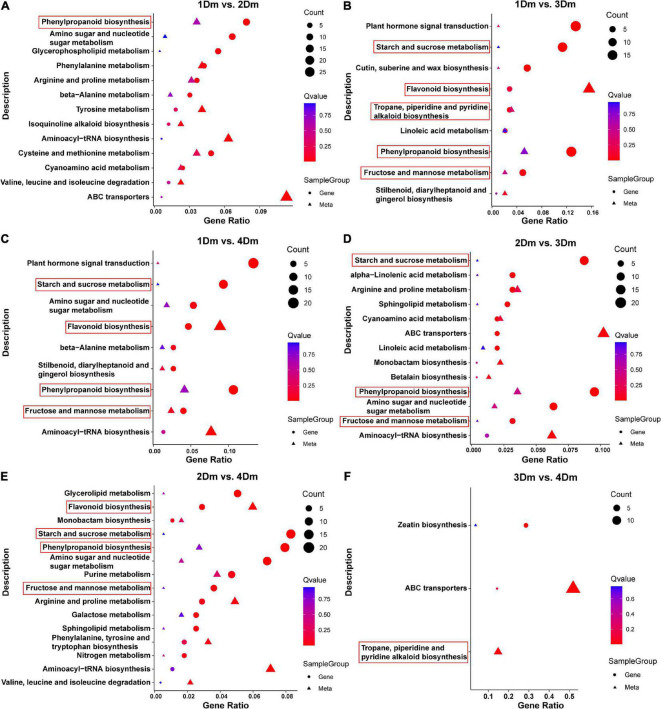
Scatterplot of KEGG pathway enrichment for transcriptome and metabolome. The circles indicate genes and the triangles indicate metabolites. The horizontal axis is the gene ratio and the vertical axis is the pathway terms. The larger the point, the greater the number of DEGs or DAMs involved. The pathways related to flavonoid, polysaccharide and alkaloid biosynthesis are marked with red boxes. **(A)** 1Dm vs. 2Dm; **(B)** 1Dm vs. 3Dm; **(C)** 1Dm vs. 4Dm; **(D)** 2Dm vs. 3Dm; **(E)** 2Dm vs. 4Dm; **(F)** 3Dm vs. 4Dm.

To assess the relationship between transcriptome and metabolome, the KEGG pathway enrichment results were integrated (Figure 4). KEGG pathways were classified into 5 categories, including Cellular Processes, Environmental Information Processing, Genetic Information Processing, Human Diseases, Metabolism, and Organismal Systems. Among them, the Metabolism class has the most genes. The results showed a high enrichment of metabolism-related pathways in all comparison groups, such as, “Flavonoid biosynthesis,” “Phenylpropanoid biosynthesis,” “Fructose and mannose metabolism,” “Tropane, piperidine and pyridine alkaloid biosynthesis” and “Starch and sucrose metabolism.” Among them, “Flavonoid biosynthesis” and “Phenylpropanoid biosynthesis,” which are involved in flavonoid synthesis, were significantly enriched in 1Dm vs. 2Dm, 1Dm vs. 3Dm, 1Dm vs. 4Dm, 2Dm vs. 3Dm, 2Dm vs. 4Dm (Figure 4). The “Fructose and mannose metabolism” and “Starch and sucrose metabolism” pathways involved in polysaccharide synthesis were significantly enriched in 1Dm vs. 4Dm, 1Dm vs. 3Dm, 2Dm vs. 3Dm, 2Dm vs. 4Dm. The “Tropane, piperidine and pyridine alkaloid biosynthesis” pathways involved in alkaloid synthesis were significantly enriched in 1Dm vs. 3Dm and 3Dm vs. 4Dm. These are all related to the synthesis of important medicinal components of *D. moniliforme*. Moreover, based on the metabolite results, it is known that flavonoid compounds were significantly higher in 3Dm and 4Dm. Meanwhile, the transcriptome results showed a more significant effect of phenylpropanoid biosynthesis in each comparison group, while the phenylpropanoid pathway is upstream of the flavonoid pathway. In summary, both DAMs and DEGs were significantly correlated with the flavonoid synthesis pathway.

## Discussion

The differential metabolites of the stems of *D. moniliforme* at four growth years were detected. A total of 480 DAMs were identified, including 107 types of flavonoid metabolites, mainly flavonoids and flavonols. It is worth noting that 3Dm and 4Dm have much higher expression levels of key flavonoids, such as eriodictyol, kaempferol, quercetin, and isorhamnetin than 1Dm and 2Dm. These metabolites have been reported to have antioxidant (19), anti-inflammatory (20), prevention and treatment of cardiovascular diseases (21), anti-tumor (22), kidney protection (23) and other medicinal properties. Compared with those of our previous studies, the numbers of species of flavonoids metabolites in *D. moniliforme* were fewer than those in *D. huoshanense* (24). The accumulation pattern of flavonoids was different in different species of *Dendrobium*. In *D. huoshanense*, flavonoids showed a tendency of accumulation, and the content of flavonoids was the highest in stems of 4-year-old, whereas in *D. moniliforme*, the content of flavonoids was the highest in 3-year-old stems, but decreased in 4-year-old stems. Meanwhile, the content of flavonoids in stems of 3-year-old *D. moniliforme* was 11.13 mg^.^g^–1^ DW, which was higher than that in stems of 4-year-old *D. huoshanense* (8.94 mg^.^g^–1^ DW). Therefore, *D. moniliforme* is more suitable as an antioxidant to scavenge free radicals in the human body than *D. huoshanense*. Meanwhile, the best effect was obtained from 3-year-old *D. moniliforme*. The results of this experiment illustrate the great potential of *D. moniliforme* as a food and cosmetic ingredient.

With the advancement of sequencing technology and the growing volume of transcriptome data, WGCNA analysis allows for the quick and efficient identification of genes or TFs associated with specific traits. In this study, the WGCNA was used to screen out a *FLS* gene (*LOC110114894*) that is highly correlated with flavonoid accumulation, regulates the biosynthesis of flavonoids, and influences the production of related secondary metabolites including kaempferol and quercetin. This is consistent with the results of previous studies with findings on the transcriptional regulation of *FLS* genes in species such as *Vitis vinifera* (25), *Camellia nitidissima* (26), and *Scutellaria baicalensis* (27), which also found that FLS is a key enzyme in the flavonol biosynthesis pathway, significantly correlated with total flavonol content, and its high expression promoted the accumulation of flavonols (28). This gene was also found in the transcriptome of *D. huoshanense*. The results of transcriptome sequencing and qRT-PCR results showed that the expression level of *FLS* in *D. moniliforme* was higher than that in *D. huoshanense*. Therefore, we hypothesized that the expression level of *FLS* affected the biosynthesis level of flavonoids in *Dendrobium.*

MYB transcription factors (TFs) are of great interest because of their importance in repressing or activating the transcription of genes related to the biosynthesis of anthocyanins, proanthocyanidins, flavonols, and other flavonoid biosynthesis in plants (29). In this study, one *MYB1R* (*LOC110103484*) and one *MYB86* (*LOC110093022*) were identified as hub genes, indicating that they play an important role in regulating flavonoid metabolism. In tobacco, *Arabidopsis* and other plants, MYB1R has been reported as transcriptional repressors of anthocyanin biosynthesis (30, 31). In our study, overexpression of MYB1R was hypothesized as one of the reasons for the deficiency of anthocyanins. Furthermore, MYB86 was found to be up-regulated in the first and second years, while down-regulated in the third and fourth years. Gao et al. found that MYB86 was up-regulated during initial fruit development, which may promote the accumulation of anthocyanin, and this may explain the up-regulation of MYB86 in the first and second years (32). It was found that cinnamoyl-CoA reductase (CCR) is the entry point of phenylpropanoid pathway into lignin pathway (33). In this study, a key enzyme involved in lignin synthesis, *CCR* (*LOC110107374*), in the turquoise module was identified, suggesting that lignin plays a key regulatory role in the synthesis of flavonoids. This has been confirmed in *Arabidopsis*, tobacco, peach and other plants (34–36).

Also, a regulatory network for flavonoid biosynthesis in *D. moniliforme* stems was built to visualize the role of flavonoid synthesis genes in the pathway (Figure 2). The synthesis of flavonoids starts from the phenylpropanoid metabolic pathway, which is one of the most well-studied secondary metabolic pathways (37). The phenylpropanoid metabolic pathway contains enzymes such as PAL, C4H, and 4CL, which catalyze the conversion of phenylalanine to coumaroyl-CoA (38). Figure 2 shows that genes *PAL, C4H, and 4CL* were significantly up-regulated in *D. moniliforme* in first and second years; meanwhile, lignin in the downstream pathway was not found. Numerous studies have shown that MYBs play an significant regulatory function in the synthesis of lignin and flavonoids (39). Zhang et al. found that most R2R3-MYB contribute to flavonoid synthesis at the expense of repressing lignin synthesis (40). A large number of MYB TFs were found in the transcriptome data. So, these MYB TFs were speculated to inhibit the production of lignin and affect the expression of genes like *PAL, C4H, and 4CL* in different growth years of *D. moniliforme*.

## Conclusion

In summary, this study revealed the changes of flavonoids and related genes in different growth years, and constructs a regulatory network of flavonoid synthesis. The results showed that *FLS* occupies an important position in the biosynthesis of flavonoids. Also, the biosynthetic pathway of flavonoids is influenced by lignin biosynthesis, and a decrease in lignin biosynthesis may provide more substrates for flavonoids. MYB TFs also have a regulatory role in the flavonoid biosynthesis pathway. Transcriptomic and metabolomic data showed a gradual increase in flavonoids in the stems of *D. moniliforme* with increasing growth years. Considering factors such as time and cost, the best harvesting period for *D. moniliforme* is the third year. This study provides important data for farmers and processors involved in the *D. moniliforme* cultivation industry and provides new theory and evidence for determining the optimal harvesting period for *D. moniliforme*.

## Data availability statement

The datasets presented in this study can be found in online repositories. The names of the repository/repositories and accession number(s) can be found in the article/Supplementary material.

## Author contributions

YY: conception and design of the research, drafting the manuscript, and revision of manuscript for important intellectual content. JZ: acquisition of data, analysis and interpretation of data, drafting the manuscript, and revision of manuscript for important intellectual content. HZ: acquisition of data. MZ: statistical analysis. SL: conception and design of the research. All authors contributed to the article and approved the submitted version.

## Footnotes

1
www.shimadzu.com.cn/

2
www.appliedbiosystems.cn/

3
www.r-project.org

4
https://www.ncbi.nlm.nih.gov/genome/69090

## Supplementary material

Supplementary Figure 1 Plant size of *Dendrobium moniliforme* from four different years.

Supplementary Figure 2 Verification of the expression patterns of RNA-seq results using qRT-PCR.

Supplementary Figure 3 Metabolomic analysis of *D. moniliforme* with different growth years. **(A)** PCA analysis of metabolomic data. **(B)** Heatmap of all metabolites detected in *D. moniliforme*. **(C)** K-means cluster analysis. **(D)** The number of up and down regulated differential metabolites in different groups. 1, 2, 3, and 4Dm represent the four growth stages of *D. moniliforme*: 1Dm represents the first year of growth; 2Dm represents the second year of growth; 3Dm represents the third year of growth; 4Dm represents the fourth year of growth.

Supplementary Figure 4 Top 10 up-accumulated and down-accumulated metabolites with significant differences. The metabolites marked in red are flavonoid metabolites. **(A)** 1Dm vs. 2Dm; **(B)** 1Dm vs. 3Dm; **(C)** 1Dm vs. 4Dm; **(D)** 2Dm vs. 3Dm; **(E)** 2Dm vs. 4Dm; **(F)** 3Dm vs. 4Dm.

Supplementary Figure 5 Differentially expressed genes (DEGs) in different comparisons. The red dots mean significantly up-regulated genes and the blue dots represent significantly down-regulated genes. The gray dots represent non-DEGs. **(A)** 1Dm vs. 2Dm volcano; **(B)** 1Dm vs. 3Dm volcano; **(C)** 1Dm vs. 4Dm volcano; **(D)** 2Dm vs. 3Dm volcano; **(E)** 2Dm vs. 4Dm volcano; **(F)** 3Dm vs. 4Dm volcano.

Supplementary Figure 6 GO enrichment circle diagram. The outermost circle indicates the GO term, and different colors represent different subcategories; the second circle indicates the number and *P*-value of that GO term. The more genes the longer the bar, the smaller the value the redder the color; the third circle indicates the number of up- and down-regulated genes of the GO term, red indicates up-regulation and green indicates down-regulation. The fourth circle indicates the Rich Factor value of each classification. **(A)** 1Dm vs. 2Dm volcano; **(B)** 1Dm vs. 3Dm; **(C)** 1Dm vs. 4Dm; **(D)** 2Dm vs. 3Dm; **(E)** 2Dm vs. 4Dm; **(F)** 3Dm vs. 4Dm.

Supplementary Figure 7 KEGG pathway enrichment bubble diagram. The bubble diagram shows the top 20 KEGG enrichment pathways. Each bubble represents a different pathway. The bubble color represents the KEGG category and the bubble size represents the number of DEGs. The smaller the *p*-value of the enriched pathway, the more significant it is. **(A)** 1Dm vs. 2Dm; **(B)** 1Dm vs. 3Dm; **(C)** 1Dm vs. 4Dm; **(D)** 2Dm vs. 3Dm; **(E)** 2Dm vs. 4Dm; **(F)** 3Dm vs. 4Dm.

Supplementary Figure 8 Top 20 KEGG pathways. **(A)** 1Dm vs. 2Dm; **(B)** 1Dm vs. 3Dm; **(C)** 1Dm vs. 4Dm; **(D)** 2Dm vs. 3Dm; **(E)** 2Dm vs. 4Dm; **(F)** 3Dm vs. 4Dm.

Supplementary Figure 9 The heatmap of the expression of hub genes in four modules. **(A)** yellow module; **(B)** brown module; **(C)** turquoise module; **(D)** black module.

Supplementary Table 1 Gene IDs and primers used in the quantitative real-time PCR (qRT-PCR) experiments.

Supplementary Tables 2–7 The complete list of identified metabolites.

## References

[1] Meng Q Fan H Chen F Xiao T Zhang L. Preparation and characterization of *Dendrobium officinale* powders through superfine grinding. *J Sci Food Agric.* (2018) 98:1906–13. 10.1002/jsfa.8672 28902405

[2] Cheng J Dang P-P Zhao Z Yuan L-C Zhou Z-H Wolf D An assessment of the Chinese medicinal *Dendrobium* industry: supply, demand and sustainability. *J Ethnopharmacol.* (2019) 229:81–8. 10.1016/j.jep.2018.09.001 30266420

[3] Shah S Shrestha R Maharjan S Selosse M-A Pant B. Isolation and characterization of plant growth-promoting endophytic fungi from the roots of *Dendrobium moniliforme*. *Plants.* (2019) 8:5. 10.3390/plants8010005 30597827 PMC6359427

[4] Ye M Hou B Luo J Yan W Liu W Ding X. Genetic diversity and conservation of the endangered herb *Dendrobium moniliforme* based on amplified fragment length polymorphism markers. *Sci Hortic.* (2015) 189:51–8. 10.1016/j.scienta.2015.03.035

[5] Teixeira da Silva JA Ng TB. The medicinal and pharmaceutical importance of *Dendrobium* species. *Appl Microbiol Biotechnol.* (2017) 101:2227–39. 10.1007/s00253-017-8169-9 28197691

[6] Liu W-H Hua Y-F Zhan Z-J. Moniline, a new alkaloid from *Dendrobium moniliforme*. *J Chem Res.* (2019) 2007:317–8. 10.3184/030823407x218048

[7] Whang SS Um WS Song I-J Lim PO Choi K Park K-W Molecular analysis of anthocyanin biosynthetic genes and control of flower coloration by flavonoid 3’,5’-hydroxylase (F3’5’H) in *Dendrobium moniliforme*. *J Plant Biol.* (2011) 54:209–18. 10.1007/s12374-011-9158-7

[8] Tsai PJ Huang WC Hsieh MC Sung PJ Kuo YH Wu WH. Flavones isolated from *Scutellariae* radix suppress *Propionibacterium* acnes-induced cytokine production in vitro and in vivo. *Molecules.* (2015) 21:E15. 10.3390/molecules21010015 26712724 PMC6273464

[9] Liu J Hefni ME Witthoft CM. Characterization of flavonoid compounds in common Swedish berry species. *Foods.* (2020) 9:358. 10.3390/foods9030358 32204535 PMC7143522

[10] Yan S Zhao T Zhang X Xing J Yadong H Chun Z. Comparison of polysaccharide and dendrobine content in Hejiang *Dendrobium* nobile at different harvesting time. *China Pharmacy.* (2018) 29:73–7.

[11] Yuan Y Zhang B Tang X Zhang J Lin J. Comparative transcriptome analysis of different *Dendrobium* species reveals active ingredients-related genes and pathways. *Int J Mol Sci.* (2020) 21:861. 10.3390/ijms21030861 32013237 PMC7037882

[12] Yang B He S Liu Y Liu B Ju Y Kang D Transcriptomics integrated with metabolomics reveals the effect of regulated deficit irrigation on anthocyanin biosynthesis in cabernet sauvignon grape berries. *Food Chem.* (2020) 314:126170. 10.1016/j.foodchem.2020.126170 31978717

[13] Kim D Langmead B Salzberg SL. HISAT: a fast spliced aligner with low memory requirements. *Nat Methods.* (2015) 12:357–60. 10.1038/nmeth.3317 25751142 PMC4655817

[14] Love MI Huber W Anders S. Moderated estimation of fold change and dispersion for RNA-seq data with DESeq2. *Genome Biol.* (2014) 15:550. 10.1186/s13059-014-0550-8 25516281 PMC4302049

[15] Feng T Li K Zheng P Wang Y Lv Y Shen L Weighted gene coexpression network analysis identified MicroRNA coexpression modules and related pathways in type 2 diabetes mellitus. *Oxid Med Cell Longev.* (2019) 2019:9567641. 10.1155/2019/9567641 31915515 PMC6935443

[16] Yip AM Horvath S. Gene network interconnectedness and the generalized topological overlap measure. *BMC Bioinformatics.* (2007) 8:22. 10.1186/1471-2105-8-22 17250769 PMC1797055

[17] Li A Horvath S. Network neighborhood analysis with the multi-node topological overlap measure. *Bioinformatics.* (2007) 23:222–31. 10.1093/bioinformatics/btl581 17110366

[18] Li H Yao W Fu Y Li S Guo Q. De novo assembly and discovery of genes that are involved in drought tolerance in Tibetan Sophora moorcroftiana. *PLoS One.* (2015) 10:e111054. 10.1371/journal.pone.0111054 25559297 PMC4283959

[19] Rossato MF Trevisan G Walker CI Klafke JZ de Oliveira AP Villarinho JG Eriodictyol: a flavonoid antagonist of the TRPV1 receptor with antioxidant activity. *Biochem Pharmacol.* (2011) 81:544–51. 10.1016/j.bcp.2010.11.004 21087598

[20] Lee JK. Anti-inflammatory effects of eriodictyol in lipopolysaccharide-stimulated raw 264.7 murine macrophages. *Arch Pharm Res.* (2011) 34:671–9. 10.1007/s12272-011-0418-3 21544733

[21] Zhang N Pei F Wei H Zhang T Yang C Ma G Isorhamnetin protects rat ventricular myocytes from ischemia and reperfusion injury. *Exp Toxicol Pathol.* (2011) 63:33–8. 10.1016/j.etp.2009.09.005 19815400

[22] Wang JL Quan Q Ji R Guo XY Zhang JM Li X Isorhamnetin suppresses PANC-1 pancreatic cancer cell proliferation through S phase arrest. *Biomed Pharmacother.* (2018) 108:925–33. 10.1016/j.biopha.2018.09.105 30372904

[23] Yamaguchi M Hamamoto R Uchiyama S Ishiyama K. Effects of flavonoid on calcium content in femoral tissue culture and parathyroid hormone-stimulated osteoclastogenesis in bone marrow culture in vitro. *Mol Cell Biochem.* (2007) 303:83–8. 10.1007/s11010-007-9458-x 17541507

[24] Yuan Y Zuo J Zhang H Li R Yu M Liu S. Integration of transcriptome and metabolome provides new insights to flavonoids biosynthesis in *Dendrobium huoshanense*. *Front Plant Sci.* (2022) 13:850090. 10.3389/fpls.2022.850090 35360302 PMC8964182

[25] Fang F Tang K Huang W-D. Changes of flavonol synthase and flavonol contents during grape berry development. *Eur Food Res Technol.* (2013) 237:529–40. 10.1007/s00217-013-2020-z

[26] Zhou XW Fan ZQ Chen Y Zhu YL Li JY Yin HF. Functional analyses of a flavonol synthase-like gene from *Camellia nitidissima* reveal its roles in flavonoid metabolism during floral pigmentation. *J Biosci.* (2013) 38:593–604. 10.1007/s12038-013-9339-2 23938391

[27] Kim YB Kim K Kim Y Tuan PA Kim HH Cho JW Cloning and characterization of a flavonol synthase gene from *Scutellaria baicalensis*. *Sci World J.* (2014) 2014:980740. 10.1155/2014/980740 24672406 PMC3927949

[28] Duan Y Eduardo Melo Santiago F Rodrigues Dos Reis A de Figueiredo MA Zhou S Thannhauser TW Genotypic variation of flavonols and antioxidant capacity in broccoli. *Food Chem.* (2021) 338:127997. 10.1016/j.foodchem.2020.127997 33091988

[29] Nabavi SM Šamec D Tomczyk M Milella L Russo D Habtemariam S Flavonoid biosynthetic pathways in plants: versatile targets for metabolic engineering. *Biotechnol Adv.* (2020) 38:107316. 10.1016/j.biotechadv.2018.11.005 30458225

[30] Zhu H-F Fitzsimmons K Khandelwal A Kranz RG. CPC, a single-repeat R3 MYB, is a negative regulator of anthocyanin biosynthesis in *Arabidopsis*. *Mol Plant.* (2009) 2:790–802. 10.1093/mp/ssp030 19825656

[31] Nakatsuka T Yamada E Saito M Fujita K Nishihara M. Heterologous expression of gentian MYB1R transcription factors suppresses anthocyanin pigmentation in tobacco flowers. *Plant Cell Rep.* (2013) 32:1925–37. 10.1007/s00299-013-1504-4 24037114

[32] Gao X Wang L Zhang H Zhu B Lv G Xiao J. Transcriptome analysis and identification of genes associated with floral transition and fruit development in rabbiteye blueberry (*Vaccinium ashei*). *PLoS One.* (2021) 16:e0259119. 10.1371/journal.pone.0259119 34710165 PMC8553168

[33] Yin N Li B Liu X Liang Y Lian J Xue Y Two types of cinnamoyl-CoA reductase function divergently in accumulation of lignins, flavonoids and glucosinolates and enhance lodging resistance in *Brassica napus*. *Crop J.* (2021) 10:647–60. 10.1016/j.cj.2021.10.002

[34] Shi J Yan X Sun T Shen Y Shi Q Wang W Homeostatic regulation of flavonoid and lignin biosynthesis in phenylpropanoid pathway of transgenic tobacco. *Gene.* (2022) 809:146017. 10.1016/j.gene.2021.146017 34655725

[35] Besseau S Hoffmann L Geoffroy P Lapierre C Pollet B Legrand M. Flavonoid accumulation in *Arabidopsis* repressed in lignin synthesis affects auxin transport and plant growth. *Plant Cell.* (2007) 19:148–62. 10.1105/tpc.106.044495 17237352 PMC1820963

[36] Dardick CD Callahan AM Chiozzotto R Schaffer RJ Piagnani MC Scorza R. Stone formation in peach fruit exhibits spatial coordination of the lignin and flavonoid pathways and similarity to *Arabidopsis* dehiscence. *BMC Biol.* (2010) 8:13. 10.1186/1741-7007-8-13 20144217 PMC2830173

[37] Fraser CM Chapple C. The phenylpropanoid pathway in *Arabidopsis*. *Arabidopsis Book.* (2011) 9:e0152. 10.1199/tab.0152 22303276 PMC3268504

[38] Moldovan B David L. Bioactive flavonoids from *Cornus mas* L. fruits. *Mini Rev Org Chem.* (2017) 14:489–95. 10.2174/1573398X13666170426102809

[39] Zhu L Shan H Chen S Jiang J Gu C Zhou G The heterologous expression of the chrysanthemum R2R3-MYB transcription factor CmMYB1 alters lignin composition and represses flavonoid synthesis in *Arabidopsis thaliana*. *PLoS One.* (2013) 8:e65680. 10.1371/journal.pone.0065680 23840353 PMC3686752

[40] Zhang S Yang J Li H Chiang VL Fu Y. Cooperative regulation of flavonoid and lignin biosynthesis in plants. *Crit Rev Plant Sci.* (2021) 40:109–26. 10.1080/07352689.2021.1898083

