# Supplementary material for: Analysis of the different growth years accumulation of flavonoids in Dendrobium moniliforme (L.) Sw. by the integration of metabolomic and transcriptomic approaches
Source: Front Nutr. 2022 Sep 26;9:928074. doi: 10.3389/fnut.2022.928074 (PMC9549206; doi:10.3389/fnut.2022.928074)
Supplement: Supplementary Figure 1 — Plant size of Dendrobium moniliforme from four different years. [file Data_Sheet_1.ZIP › Supplementary table S1.docx]

**Table S1** **Genes IDs and primers used in the quantitative real-time PCR (qRT-PCR) experiments.**

| Primer_ID | Forward PCR Primer (5′-3′) | Reverse PCR Primer (5′-3′) |
| --- | --- | --- |
| *Actin* | TCCCAAGGCAAACAGAGAAA | GGCCACTAGCATATAGGGAAAG |
| LOC110097226 (*4CL*1)  LOC110115146 (*4CL*2)  LOC110109032 (*4CL*3)  LOC110113575 (*C4H*)  LOC110099164 (*CHI*)  LOC110113809 (*CHS*1)  LOC110115249 (*CHS*2)  LOC110105073 (*CHS*3)  LOC110105791 (*CHS*4)  LOC110113094 (*F3'5'H*1)  LOC110106061 (*F3'5'H*2)  LOC110106800 (*F3H*)  LOC110114894 (*FLS*1)  LOC110097028 (*FLS*2)  LOC110096779 (*F3'H*1)  LOC110109131 (*F3'H*2)  LOC110115785 (*PAL*) | TGATTTCCGTTGCCTCACCCGAT  CTTTTCTCCAAACCGCACA  TAAACCCCTTCACCGCTGCC  TCGCATCCCTGGCAAAGAAGTTCG  CTGCCATTAACGGGTAAACAGT  GTGCTCGCCATCGGAACCTC  ATGCCGAGCCTTGAATCCA  ATGCCGAGCCTTGAATCCA  CGGCCAACCCTCCCAATGC  CAGTTGCCACCATCATACTCT  TCTCCGATGTTGTTCAATCCC  CCAGTGCAAAATGCCTTCGTT  GCGTCCAATCCCTTTCCGAT  GTACAGAGAGTTCAATCCATCGC  CTTAGCACCAATACTCTTGTCA  ACTCTACAATCTTCGCGCTCT  TCTCACCGCCGCACAAAGC | CCCAGCTACGGAGTTTGTCCAC  CAATTTGGAGGCACAATAGCA  CTTTGCCACATAATCCATCACCT  GTATATGGAGACTGAACTGCCCTC  ATTCCAATACCGCATTCGTCA  ACCGCACCAGCAATCGGAA  CTCCTGAATTGCCTTGGTTG  GCCCAGCTTTGGTATCTCCC  CCAAACGAAATGGCGCTTCCT  GGATCTCTGTGAATTGCCCAT  TGGCCCACACATTTACCAG  ACCGTTGCTCAAGTAATGACC  CGATCTTGGCTCACTAGTTCCG  CAGGCCCCATAATTAATGCT  CTTGAACTCACTCGGGTCA  ACCATGCGACTGATGACGTT  AGGCGAACACATACTCCCT |
