# Supplementary figures and images for: Analysis of the different growth years accumulation of flavonoids in Dendrobium moniliforme (L.) Sw. by the integration of metabolomic and transcriptomic approaches
Source: Front Nutr. 2022 Sep 26;9:928074. doi: 10.3389/fnut.2022.928074 (PMC9549206; doi:10.3389/fnut.2022.928074)

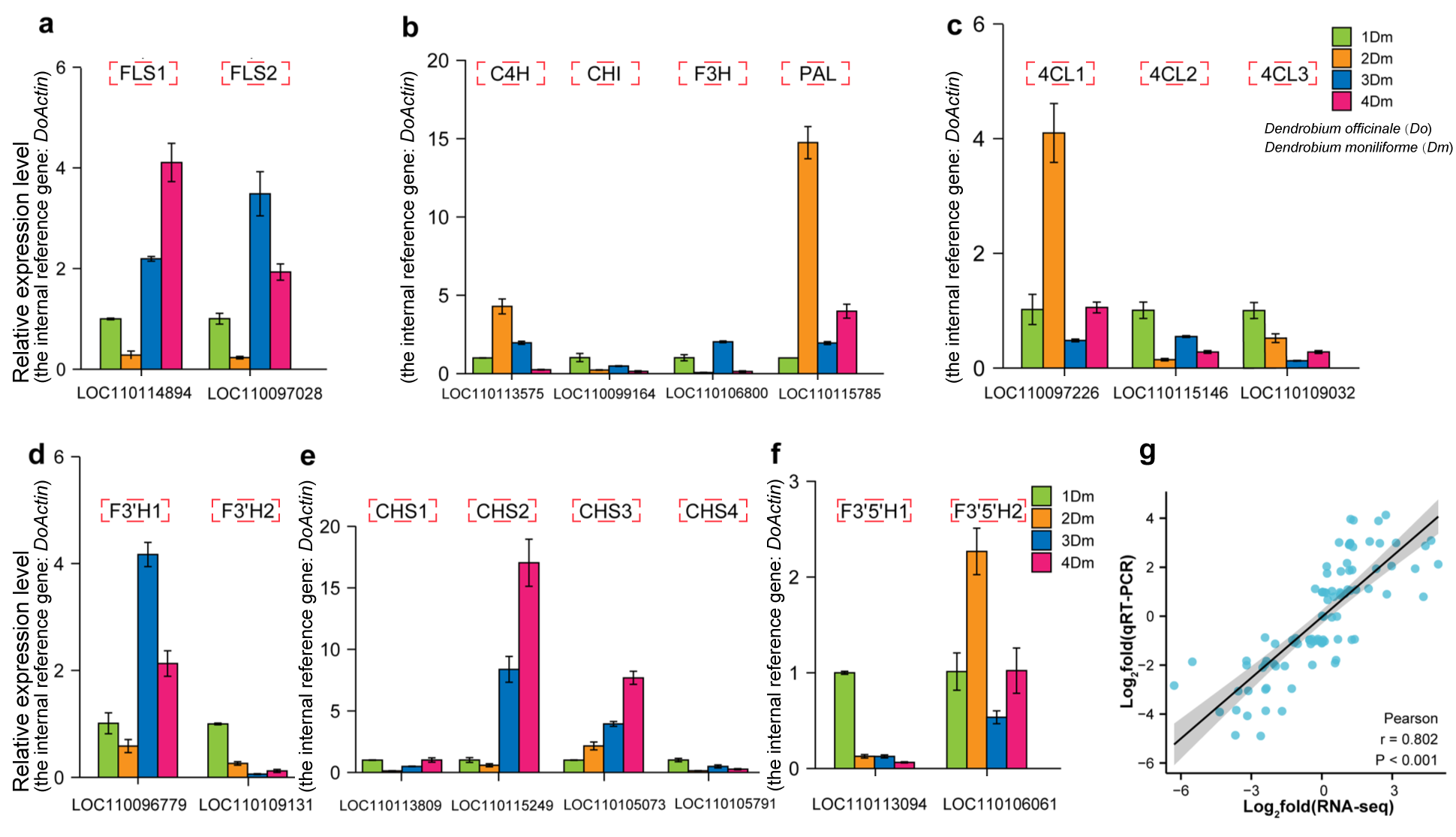

Supplement: Supplementary Figure 1 — Plant size of Dendrobium moniliforme from four different years. [file Data_Sheet_1.ZIP › Figure S2.tif]

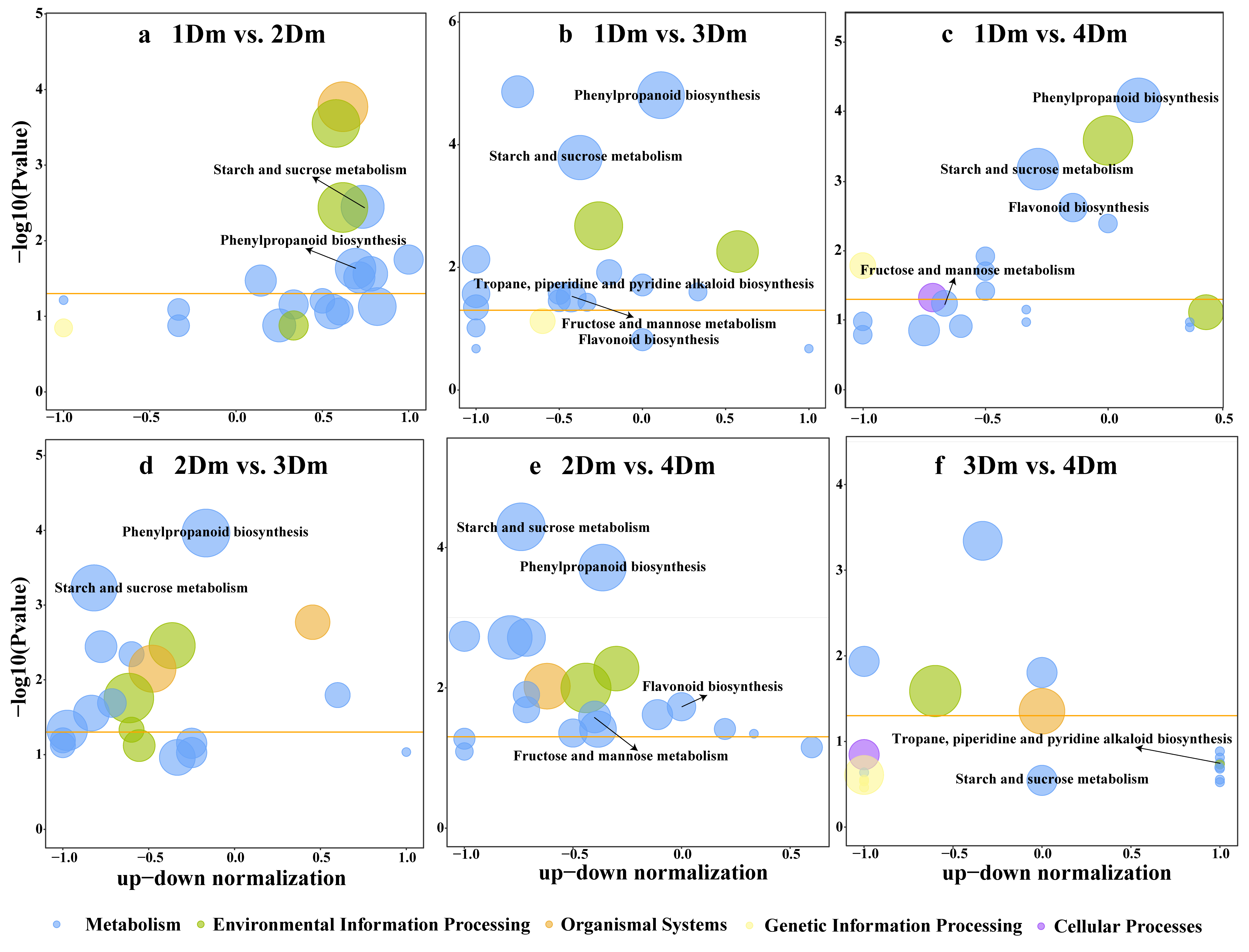

Supplement: Supplementary Figure 1 — Plant size of Dendrobium moniliforme from four different years. [file Data_Sheet_1.ZIP › Figure S7.tif]

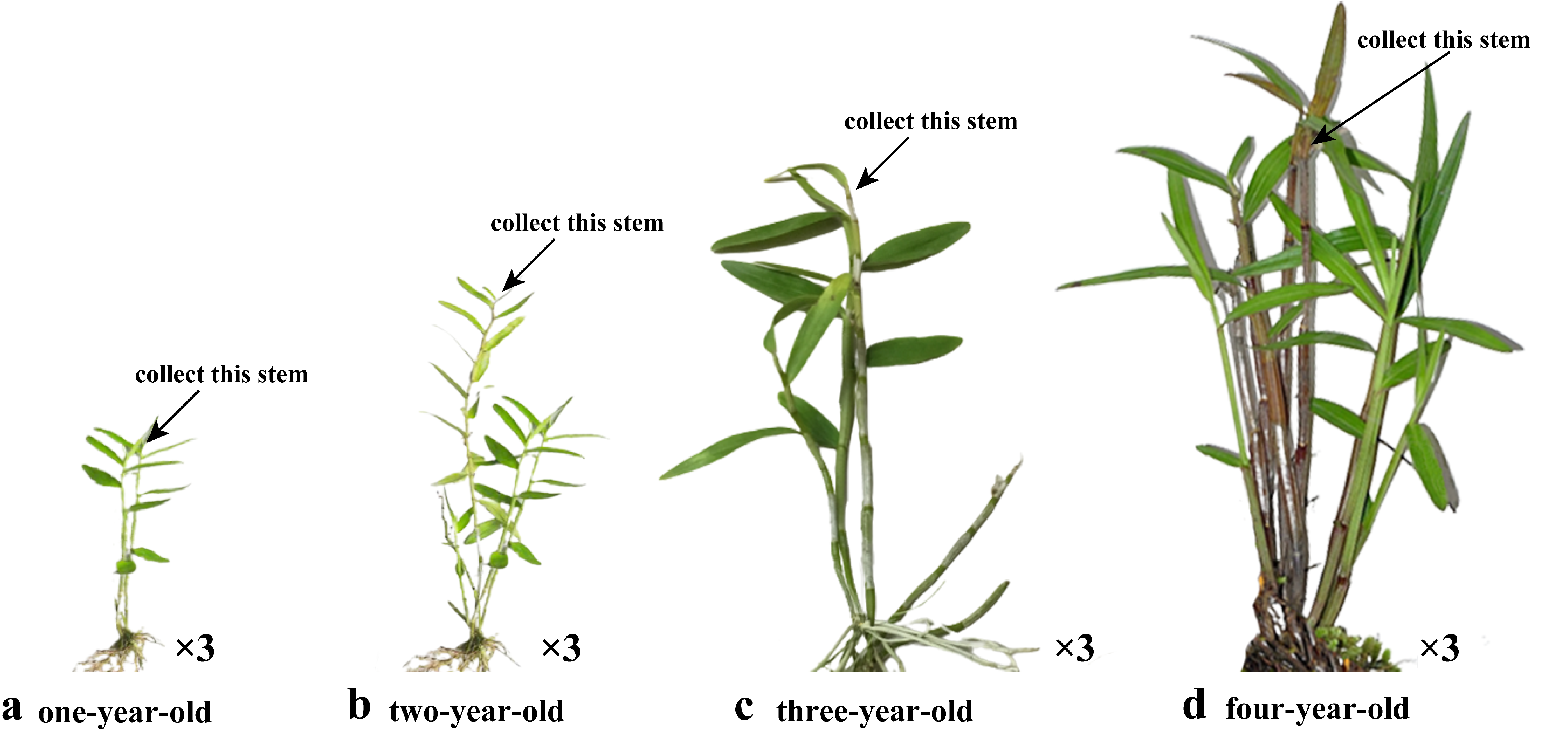

Supplement: Supplementary Figure 1 — Plant size of Dendrobium moniliforme from four different years. [file Data_Sheet_1.ZIP › figure S1.tif]
